# Short-wavelength plasma turbulence and temperature anisotropy instabilities: recent computational progress

**DOI:** 10.1098/rsta.2014.0149

**Published:** 2015-05-13

**Authors:** S. Peter Gary

**Affiliations:** Space Science Institute, Boulder, CO, USA

**Keywords:** turbulence, instabilities, simulations

## Abstract

Plasma turbulence consists of an ensemble of enhanced, broadband electromagnetic fluctuations, typically driven by multi-wave interactions which transfer energy in wavevector space via non- linear cascade processes. Temperature anisotropy instabilities in collisionless plasmas are driven by quasi-linear wave–particle interactions which transfer particle kinetic energy to field fluctuation energy; the resulting enhanced fluctuations are typically narrowband in wavevector magnitude and direction. Whatever their sources, short-wavelength fluctuations are those at which charged particle kinetic, that is, velocity-space, properties are important; these are generally wavelengths of the order of or shorter than the ion inertial length or the thermal ion gyroradius. The purpose of this review is to summarize and interpret recent computational results concerning short-wavelength plasma turbulence, short-wavelength temperature anisotropy instabilities and relationships between the two phenomena.

## 1. Introduction

Plasma turbulence is an ensemble of large-amplitude, broadband and incoherent electromagnetic field and particle fluctuations. Plasma turbulence at long wavelengths is usually adequately described by magnetohydrodynamics (MHD) fluid models. As in neutral fluid turbulence models, such theories predict that nonlinear multi-wave processes carry fluctuating energy injected at long wavelengths through decreasing wavelengths to eventual dissipation, that is, conversion to thermal energy, at sufficiently short scale lengths. This forward cascade in homogeneous plasmas described by MHD yields magnetic fluctuation spectra of the form *E*(*k*)∼*k*^−5/3^ [1] with a strong wavevector anisotropy such that *k*_⊥_≫*k*_∥_, where the two subscripts represent directions, respectively, perpendicular and parallel to the background uniform magnetic field **B**_o_ [2].

In MHD and other fluid models of plasma turbulence, dissipation at short wavelengths is typically assumed to be provided by collisional processes such as resistivity and/or viscosity. But in collisionless plasmas such as the solar wind, the medium which currently provides the best opportunity for the study of homogeneous plasma turbulence, particle–particle collisions are weak and plasma processes involving relatively short-wavelength fluctuations make important contributions to the dissipation. Both observations and kinetic simulations have demonstrated that the MHD description of plasma turbulence fails at fluctuation wavelengths of the order of or shorter than either the thermal ion gyroradius or the ion inertial length. Here we define the ion (electron) inertial length λ_i_=*c*/*ω*_pi_(λ_e_=*c*/*ω*_pe_), where *ω*_p*j*_ is the plasma frequency of the *j*th species, and the ion (electron) thermal gyroradius *ρ*_i_=*v*_i_/*Ω*_i_(*ρ*_e_=*v*_e_/*Ω*_e_), where *v*_*j*_ is the thermal speed of the *j*th species, and *Ω*_*j*_ is the cyclotron frequency of the *j*th species. Other symbols used here include *θ* as the fluctuation propagation angle (i.e. 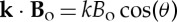) and the *j*th species parallel beta 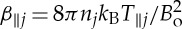. We use the wavevector anisotropy factor defined by Shebalin *et al.* [2]
1.1
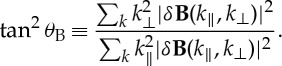


Measurements of turbulent magnetic fluctuations in the solar wind indicate that, on the observed frequency range 10^−4^ Hz<*f*<0.5 Hz, turbulent spectra typically scale as *f*^−5/3^; this is called the ‘inertial range’. Under the assumption that the phase speed of the fluctuations is very small compared with the magnitude of the solar wind flow velocity **v**_sw_, the Taylor hypothesis that 2*πf*≅**k**⋅**v**_sw_ implies that inertial range magnetic spectra scale as *k*^−5/3^. Near observed frequencies of about 0.5 Hz, measurements usually show a spectral break to *k*^−*α*^ with *α*>2 at shorter wavelengths. Under the Taylor hypothesis, this transition to a steeper slope approximately corresponds to *kc*/*ω*_pi_∼1; as this corresponds to fluctuations which are not well described by MHD, this spectral break is interpreted to demarcate the transition to a short-wavelength regime in which a kinetic, that is, velocity-space, description becomes necessary to represent the physics of the plasma fluctuations. We therefore define short-wavelength field fluctuations to span the frequency/wavenumber regime from the inertial range spectral break through monotonically decreasing amplitudes to higher frequencies and shorter wavelengths at which spacecraft measurements can no longer resolve physically meaningful spectra, typically in the regime of several hundred hertz (e.g. fig. 3 of [3]). Note that our definition of turbulence includes all broadband fluctuations, whether they arrive at the short-wavelength regime by the classic forward cascade of multi-wave interactions or by other more general nonlinear processes such as those which act in the particle-in-cell (PIC) simulations of Karimabadi *et al.* [4].

In addition to the fully nonlinear processes which carry fluctuating field energy from long wavelengths to the short-wavelength regime defined above, kinetic plasma instabilities can, through linear growth and quasi-linear saturation, directly excite enhanced fluctuations in the short-wavelength regime. Plasma instabilities arise when an ionized medium undergoes a sufficiently large departure from an isotropic, homogeneous, thermal equilibrium. Under collision-dominated conditions, particle–particle interactions lead the plasma back towards equilibrium conditions. But collisions are weak in collisionless plasmas, and the task of attempting to return such a medium towards a quiescent thermal state falls to the electric and magnetic field fluctuations. A sufficiently large plasma species anisotropy, for example, will lead to the growth of the fields; the resulting enhanced fluctuations will interact strongly with that species, diminishing (but not eliminating) its anisotropy as that component is driven towards its equilibrium state.

The plasma instability ‘zoo’ is populated with hundreds of different ‘animals’. Here, we broadly classify these growing modes as either fluid-like or kinetic. The former modes are described by fluid theories such as MHD; these instabilities are often driven by spatial gradients in the plasma fluid parameters, and usually arise at wavelengths long compared with the ion thermal gyroradius or inertial length. The latter modes are driven by velocity-space anisotropies, require velocity-space descriptions, at least for the species directly responsible for instability growth, and typically have maximum growth rates arising in the short-wavelength regime. Limitations on the length of this review require that we do not consider instabilities driven by the relative flow of plasma components (e.g. ion/ion and electron/electron instabilities summarized in ch. 8 of Gary [5]). Rather, we here emphasize instabilities driven by a species temperature anisotropy (e.g. the growing modes catalogued in ch. 7 of Gary [5]). To keep things simple, we assume a homogeneous, collisionless, magnetized plasma with a single ionic species and a single electron component, both of which are represented by a single bi-Maxwellian velocity distribution with possibly different temperatures *T*_⊥_ and *T*_∥_. In addition most of the simulations considered here are initial-value problems; the instability runs are driven by an initial anisotropy on a plasma species, and the turbulence runs are driven by an initial array of enhanced fluctuations. Different conclusions may be drawn from computations which are continuously driven, but such simulations are, for the most part, beyond the purview of this review.

Given these multiple constraints, there are four types of instabilities which may arise, as follows.

### (a) Electron temperature anisotropy instabilities (*T*_⊥e_/*T*_∥e_>1)

The primary unstable mode in this case is the whistler anisotropy instability. Kinetic linear dispersion theory ([5], §7.3.2) predicts that, at *β*_∥e_>0.025, this anisotropy gives rise to growing electromagnetic fluctuations with right-hand polarized whistler dispersion and maximum growth rate at **k**×**B**_o_=0 and *kc*/*ω*_pe_<1. At smaller values of *β*_∥e_ the maximum growth rate shifts to oblique propagation with *θ*≅50^o^ and shorter wavelengths (*kc*/*ω*_pe_>1) with substantial electrostatic, as well as electromagnetic, components [6,7].

### (b) Electron temperature anisotropy instabilities (*T*_⊥e_/*T*_∥e_<1)

For this anisotropy, kinetic linear dispersion theory predicts that two distinct instabilities may grow [8–10]. The non-resonant electron firehose instability has maximum growth rate at **k**×**B**_o_ = 0 and *kc*/*ω*_pe_≪1. The *ω*_r_ versus *k*_∥_ dispersion plot is a highly sensitive function of the anisotropy, and for the unstable regime the mode is left-hand circularly polarized at parallel or antiparallel propagation ([5], §7.3.1). The resonant electron firehose instability has zero real frequency but maximum growth rates at propagation oblique to **B**_o_ and *kc*/*ω*_pe_≪1. Both modes require *β*_∥e_>1 to show appreciable growth rates.

### (c) Ion temperature anisotropy instabilities (*T*_⊥i_/*T*_∥i_>1)

In this case, kinetic linear dispersion theory predicts that two electromagnetic instabilities may arise ([5], §7.2). The Alfvén-cyclotron instability has Alfvén-cyclotron mode dispersion with 0<*ω*_r_<*Ω*_i_, maximum growth rate at **k**×**B**_o_=0 and *kc*/*ω*_pi_<1. The ion mirror instability satisfies *ω*_*r*_ = 0 in a homogeneous plasma with maximum growth rate at propagation substantially oblique to **B**_o_ but also *kc*/*ω*_pi_<1. Kinetic linear theory predicts that the ion mirror instability has the lower threshold anisotropy at *β*_∥i_ values greater than order unity, whereas the Alfvén-cyclotron instability has the lower threshold at smaller values of *β*_∥*i*_ [11].

### (d) Ion temperature anisotropy instabilities (*T*_⊥i_/*T*_∥i_<1)

Here, also, kinetic linear theory predicts that two electromagnetic instabilities may be excited. The parallel firehose instability satisfies magnetosonic-whistler-like dispersion (fig. 7.1 of Gary [5]) with maximum growth rate at **k**×**B**_o_=0 and *kc*/*ω*_*pi*_<1. The oblique firehose instability satisfies *ω*_r_=0 in a homogeneous plasma with maximum growth rate at propagation substantially oblique to **B**_o_ but also *kc*/*ω*_pi_<1. Both firehose modes require *β*_∥*i*_>1 to exhibit appreciable growth, and linear theory predicts that the two modes may have competitive growth rates for a range of parameters near threshold [12]. (For an alternative summary of the four ion temperature anisotropy instabilities, see Howes [13].)

For plasma fluctuations described by a weak turbulence fluid model, the fundamental processes of energy transfer are nonlinear multi-wave interactions. The most basic of these is the three-wave interaction; the equations which represent conservation of fluctuation energy and momentum in such coupling [2] are
1.2
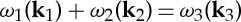
and
1.3
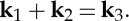
In fluid models, the forward and inverse cascade processes of turbulence require both of these conditions to be (approximately) satisfied for a broad range of wavevectors and frequencies. But three-wave interactions cannot heat particles or damp fluctuations in collisionless plasmas. To achieve the transfer of energy between field fluctuations and plasma particles, more complete kinetic descriptions of collisionless plasma dynamics are required. Linear kinetic theory [5] and associated quasi-linear formalisms involve wave–particle interactions which are strongest when the conditions of Landau resonance
1.4
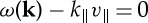
and/or cyclotron resonance
1.5
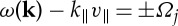
in a magnetized plasma are satisfied. If the *j*th species velocity distribution has an appreciable value at parallel particle velocity *v*_∥_ which satisfies either equation (1.4) or equation (1.5), that species is said to be in resonance with the fluctuation corresponding to *ω*(**k**), and there can be a strong exchange of energy between that fluctuation and particles with that velocity. Wave–particle interactions which cause plasma species to gain energy correspond to wave damping, whereas wave–particle interactions which transfer energy from particles to fluctuating fields are sources of plasma instabilities. Nonlinear wave–particle interactions involve the interactions of plasma particles with two or more fluctuations; for example, the condition for the two-wave nonlinear Landau resonance is *ω*_1_(**k**_1_)−*ω*_2_(**k**_2_)=(*k*_∥1_−*k*_∥2_)*v*_∥_.

In descriptions of plasma turbulence, the emphasis is often on multi-wave interactions and the associated transfer of energy or ‘cascade’ from one wavevector regime to another. In the wave–particle picture, the emphasis is usually on fluctuation damping and plasma instabilities, and the transfer of energy between waves and particles. The primary purpose of this review is to summarize and interpret recently published papers describing computational studies which illuminate the relationships between these two distinct phenomena for short-wavelength enhanced fluctuations in collisionless plasmas. The way we do this is, for each of the sub-categories listed above, to first summarize turbulent computations, then to review instability simulations, and finally to interpret the relationship between the two and to pose relevant questions which may be addressed by future studies. I agree with Howes [13] that there is a mismatch between the spectral properties of broadband turbulence and narrowband fluctuations driven by kinetic instabilities. However, I disagree with the Howes [13] conclusion that the instability-driven fluctuations ‘… merely persist alongside the anisotropic … turbulence’. In my opinion, definitive PIC simulations of possible interactions between cascading turbulence and enhanced instability-driven fluctuations have not yet been carried out. I hope the questions posed below will lead to a more complete understanding of the relationship between these two distinct phenomena.

PIC simulations compute the velocity-space dynamics of ions and electrons using the equations of motion of superparticles self-consistently coupled to the Maxwell equations representations of the electric and magnetic fields. Among the various types of simulations used to study collisionless plasmas, PIC simulations provide the most general representation of plasma dynamics, but also require the greatest computational resources in terms of capacity and run times. Full three-dimensional simulations of whistler turbulence are now being carried out [14–18] because they require relatively small computational domains and modest run times, but three-dimensional PIC simulations of longer wavelength turbulence (e.g. [4]) are much more demanding and at the time of writing remain relatively rare.

Hybrid simulations provide a full kinetic representation of ion dynamics, but use one of several fluid models to describe electron physics. Thus, hybrid simulations are expected to provide a substantially accurate picture of turbulence at kinetic ion wavelengths, that is, of the order of the ion gyroradius and ion inertial length, but become less accurate at shorter wavelengths where electron kinetic physics becomes important. But because hybrid simulations are much less demanding of computational resources than PIC codes, they have been frequently used to study electromagnetic ion instabilities and plasma turbulence at kinetic ion wavelengths.

Gyrokinetic simulation codes are based on gyrokinetic theory, a low-frequency anisotropic limit of kinetic theory which averages out charged particle cyclotron motion. Gyrokinetic theory and gyrokinetic codes exclude fast magnetosonic waves and cyclotron resonances but retain finite Larmor radius effects and the collisionless Landau resonances [19,20]. So, although gyrokinetic codes are limited to *ω*_r_<*Ω*_*i*_, they can represent fluctuations with *k*_⊥_*ρ*_i_ much larger than unity, and therefore can represent short-wavelength turbulence due to kinetic Alfvén waves.

Finite resources limit the computing power available for turbulence computations, so that, in many simulations with full three-dimensional velocities, spatial variations are limited to two dimensions; we refer to such simulations as ‘two-dimensional’ or ‘2D’ (although some refer to such calculations as ‘2.5D’). Following general practice, we use ‘three-dimensional’ or ‘3D’ to denote simulations describing fully 3D velocity and spatial variations. For 2D simulations, it is important to distinguish among ‘in-plane’ 2D computations in which **B**_o_ is parallel to one of the two spatial coordinates; ‘inclined plane’ simulations in which **B**_o_ is at an oblique angle, neither parallel nor perpendicular, to the plane of spatial variations; and ‘out-of-plane’ simulations in which **B**_o_ is strictly perpendicular to the simulation plane. The last two types of simulations exclude or restrict the propagation of fluctuations with *k*_∥_≠0, and so exclude most wave–particle interactions which involve a non-zero parallel wavenumber (e.g. equations (1.4) and (1.5)). Inasmuch as Landau and cyclotron resonances are the primary drivers of the kinetic instabilities, as well as important sources of turbulent dissipation, our emphasis here is on the ‘in-plane’ 2D simulations. We mention representative simulations which have addressed short-wavelength turbulence by ‘out-of-plane’ 2D simulations [21–25] and by steeply ‘inclined plane’ 2D simulations [26] without further comment.

## 2. Whistler turbulence and whistler anisotropy instabilities

### (a) Whistler turbulence simulations

The several PIC computational studies of whistler turbulence have assumed a homogeneous, collisionless, magnetized plasma on which an initial ensemble of relatively long-wavelength fluctuations are imposed; the individual modes satisfy the predictions of kinetic linear theory for whistler fluctuations. Gary *et al.* [27], Saito *et al.* [28,29], Saito & Gary [30] and Saito & Nariyuki [31] carried out 2D PIC simulations in which the background magnetic field **B**_o_ lies in the simulation plane, whereas full 3D PIC simulations of whistler turbulence were carried out by Chang *et al.* [14–16] and Gary *et al.* [17]. In each of these studies, a spectrum of whistlers (over 0.10<*kc*/*ω*_pe_<0.40) were imposed as an initial condition, and the fluctuations freely decayed in time. These simulations considered variations in both *β*_e_ and the initial fluctuation amplitudes; in all cases, the 2D simulations yielded results qualitatively equivalent to those of the 3D computations. Thus in both in-plane 2D and full 3D simulations, a forward cascade from the initial spectra produced a late-time broadband spectrum of turbulence extending down to an electron dissipation range at 1.0 < *kc*/*ω*_pe_. Furthermore, the cascaded fluctuations developed characteristic wavevector anisotropies with *k*_⊥_≫*k*_∥_, although the magnitude of this anisotropy decreases with increasing *β*_e_ [15,29]. Such anisotropies are consistent with the predictions of electron magnetohydrodynamics (EMHD) models, from both analytic theory [32] and computations [33]. Kinetic effects lead to dissipation of whistler turbulence; both in-plane 2D [28,30] and 3D [17] simulations show that the short-wavelength, anisotropic fluctuation spectra induced by the whistler forward cascade produce preferential heating of electrons in *T*_∥e_, consistent with the interpretation that the Landau wave–particle resonance is the primary channel of dissipation. Saito & Nariyuki [31] showed that whistler turbulence can also heat ions in directions perpendicular to **B**_o_, although ion heating by high-frequency whistlers is weaker than the corresponding electron heating [18]. The 3D simulations of Gary *et al.* [17] showed that magnetic fluctuations from the forward cascade of decaying whistler turbulence at *β*_e_=0.10 displayed a distinct spectral break at *k*_⊥_*c*/*ω*_pe_∼1. Chang *et al.* [16] demonstrated that, for relatively small levels of the initial fluctuation energy, linear collisionless damping provides most of the dissipation of the turbulence, but, as the initial fluctuation energy increases, nonlinear processes become stronger.

### (b) Whistler anisotropy instability simulations

Many PIC simulations of the whistler anisotropy instability have been run in one-dimensional (1D) [34,35] and in-plane 2D [6,36,37] configurations. These simulations show that this instability exhibits the typical quasi-linear response: exponential growth of field fluctuations to eventual saturation and the associated scattering of electrons yielding a gradual reduction of *T*_⊥e_/*T*_∥e_.

**Figure 1.**
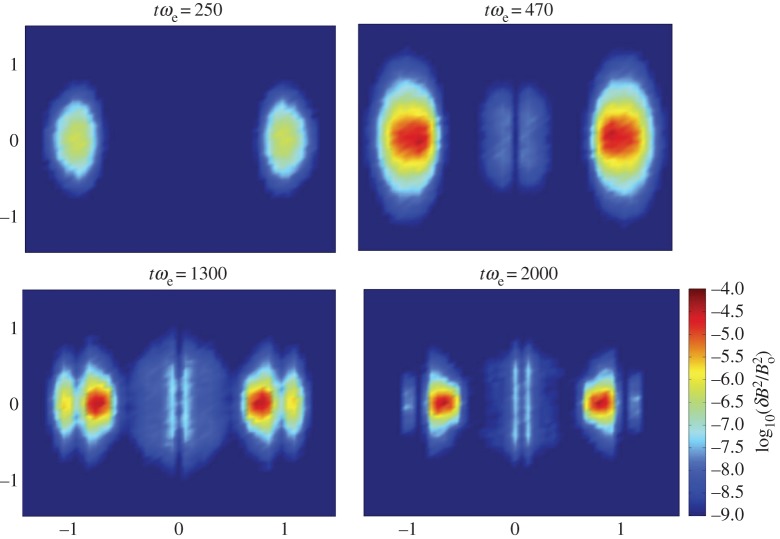
Reduced magnetic fluctuation energy spectra in (*k*_∥_,*k*_⊥_) space from a PIC simulation of the whistler anisotropy instability at four times as labelled [38]. Initial conditions here include *β*_∥e_=0.10 and *T*_⊥e_/*T*_∥e_=3.0. (Online version in colour.)

However, some recent whistler instability simulations go beyond the quasi-linear picture and illustrate the temporal enhanced magnetic fluctuation energy spectra as functions of both *k*_∥_ and *k*_⊥_. The 3D PIC simulation of Gary *et al.* [38] addresses the whistler anisotropy instability at *β*_∥*e*_=0.10, where the maximum growth rate is at parallel propagation. Figure 1 shows the resulting reduced magnetic fluctuation spectra obtained by summing over one of the two perpendicular wavevector components. During the exponential temporal growth phase (0<*ω*_pe_*t*<500), the magnetic spectrum is what one would expect; the growing fluctuations show narrowband spectra with maximum amplitudes at *k*_∥_*c*/*ω*_pe_≅1 and with *k*_⊥_≪*k*_∥_. At saturation (*ω*_pe_*t*≅470) and afterward, there is little evidence of a forward transfer of fluctuation energy, because electron cyclotron damping becomes very strong at shorter wavelengths. By contrast, there is a strong inverse transfer of fluctuation energy to small *k*_∥_, leading to distinct narrowband peaks in the parallel wavevector spectra at both *k*_∥_*c*/*ω*_pe_≅1 and *k*_∥_*c*/*ω*_pe_≪1. However, there is little extension of fluctuation energy in perpendicular wavevectors. This result is consistent with the spectral transfer being due to nonlinear three-wave interactions which satisfy equation (1.3); if both **k**_1_ and **k**_2_ have small perpendicular components, then **k**_3_ must also have a small perpendicular component. Gary *et al.* [38] further uses an argument based upon equations (1.2) and (1.3) to show that the inverse transfer is consistent with a prediction of nonlinear three-wave coupling theory.

The 2D in-plane PIC simulations of Schriver *et al.* [39] address a magnetospheric condition of several electron components in which the whistler instability is driven by a warm (*T*∼300 eV) anisotropic electron component with relatively small *β*_∥_ so that the maximum growth rate of the whistler anisotropy instability (as predicted by linear theory) arises at *θ*∼40^*o*^ and real frequencies *ω*_r_>*Ω*_*e*_/2. The simulations of Schriver *et al.* [39] further demonstrate that, at late (post-saturation) times, three-wave coupling gives rise to a range of whistler fluctuations also at oblique propagation but with *ω*_r_<*Ω*_e_/2.

Ganguli *et al.* [40] has also carried out PIC simulations of electron anisotropy instabilities, but in a more complex configuration. Their computations are run in a 2D electromagnetic PIC model in which **B**_o_ is inclined at an angle of 60° to the simulation plane. The free energy to drive the instability is provided by a ring-beam electron component at 25% of the total electron density; the ring character drives the whistler anisotropy instability with maximum growth at **k**×**B**_o_=0 and *kc*/*ω*_pe_∼1 and the beam character enables the growth of highly oblique waves near harmonics of the electron cyclotron frequency. The whistler instability drives early time narrowband enhanced fluctuations at *k*_∥_*c*/*ω*_pe_≅1; after saturation, nonlinear scattering through the Landau resonance of beat waves with the electrons, that is, through *ω*_1_−*ω*_2_=(*k*_∥1_−*k*_∥2_)*v*_∥_, allows the development of low-frequency magnetosonic/lower-hybrid waves at much smaller values of parallel wavenumbers but, again, the spectra remain narrowband.

### (c) Interpretations and questions

(1) As mentioned in §2a, the *k*_⊥_≫*k*_∥_ wavevector anisotropy induced by the forward whistler cascade leads to Landau damping and preferential heating of electrons in directions parallel and antiparallel to **B**_o_. PIC simulations of the whistler anisotropy instability [7] at *β*_∥e_>0.025 yield the opposite anisotropy of *k*_⊥_≪*k*_∥_, favouring cyclotron resonant interactions, so that pitch-angle scattering reduces *T*_⊥e_ and enhances *T*_∥e_. Finally, PIC simulations of the same instability at *β*_∥e_<0.025 give *k*_⊥_∼*k*_∥_; here also Landau damping dominates so that once again there is preferential heating of electrons in *T*_∥e_. Even though the wavevector anisotropy is very different in each of the three cases, the net result of preferential parallel heating on the electrons is the same. Can the signatures of these three distinct processes be discerned in solar wind electron observations?
(2) All of the whistler anisotropy instability simulations discussed in §2b yield narrowband spectra of fluctuations. But these spectra are very different from the broadband turbulence at the very short wavelength of electron scales as recently observed in the solar wind [3,41,42]. Therefore, if whistlers are to contribute to observed electron scale turbulence, they are less likely to be driven by instabilities than by compressive (magnetosonic-whistler) broadband turbulence at *ω*_pi_<*kc*≪*ω*_pe_. But simulations have not yet demonstrated that inertial range turbulence, of either Alfvénic or magnetosonic character, can become a source of whistler turbulence. Can simulations, either PIC or hybrid, demonstrate the possibility of such a source?
(3) Until recently, an implicit assumption of researchers using PIC simulations to study whistler turbulence has been that such high-frequency fluctuations heat only electrons. Saito & Nariyuki [31] have demonstrated that whistler fluctuations can indeed heat ions as well as electrons and have opened the door to comparative studies of species heating by short-wavelength turbulence. Relevant questions are: how do the rates of electron and ion heating by whistler turbulence scale with (*a*) the size of the simulation box and the associated turbulence wavelengths [18], (*b*) the values of *β*_e_ and *β*_i_, and (*c*) the amplitudes of the fluctuations?
(4) At *β*_e_=0.10, a 3D PIC simulation of decaying whistler turbulence by Gary *et al.* [17] showed a spectral break at a perpendicular wavenumber proportional to the inverse electron inertial length, not the inverse thermal electron gyroradius. Chen *et al.* [43] recently reported a study of proton kinetic range spectral breaks measured in the solar wind; at *β*_i_≪1 this break wavenumber scales as the inverse ion inertial length, but at *β*_i_≫1 this break wavenumber scales as the inverse thermal ion gyroradius. (See [44] for other citations concerning this topic.) This suggests an analogy with the whistler-scale break: at *β*_e_≫1 it should scale with the inverse thermal electron gyroradius. Can this be tested with a high-beta PIC simulation of decaying whistler turbulence?

## 3. Electron firehose (*T*_⊥e_<*T*_∥e_) instabilities

### (a) Electron firehose instability simulations

Significant growth of the electron firehose instabilities driven by *T*_⊥e_<*T*_∥e_ requires the electron component *β*_∥_ to be greater than unity ([5], fig. 7.8). As this condition is relatively rare for the core+halo model of electron distributions in the solar wind near 1 AU [45], most simulations of this instability to date have used a single bi-Maxwellian velocity distribution as the initial condition driving the unstable modes. Using such a model Messmer [46] and Paesold & Benz [47] carried out 1D PIC simulations of the non-resonant electron firehose instability, whereas more recent 2D PIC simulations [8–10] have compared the properties of both the non-resonant and the resonant firehose growing modes. The latter computations again demonstrate the usual quasi-linear response: exponential growth of the field fluctuations to saturation, with an associated reduction of the electron anisotropy. The most unstable fluctuations at early times are the resonant firehose modes with zero real frequency and oblique propagation; however, at late post-saturation times, propagating whistler waves emerge. Just as for the whistler anisotropy instability driven by the opposite electron anisotropy, the late-time fluctuation spectra remain relatively narrowband in wavenumber and propagation direction [10].

## 4. Alfvénic turbulence and *T*_⊥i_>*T*_∥i_ instabilities

### (a) Alfvénic turbulence simulations

**Figure 2.**
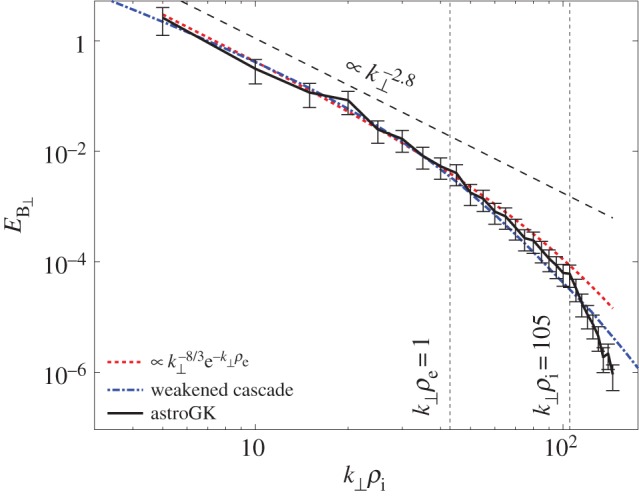
Results of a gyrokinetic simulation with *β*_i_=*β*_e_. 1D magnetic energy spectra as functions of perpendicular wavenumber. The solid black line represents results from the simulation; the dashed-dotted and dotted lines represent two model predictions and the dashed line represents a fixed spectral index of −2.8. (Adapted with permission from [49].) (Online version in colour.)

A substantial body of computational research on Alfvénic turbulence is the work of Howes and collaborators using a 3D gyrokinetic code. The simulations of Howes and co-workers [19,20,48–50] assume a homogeneous, weakly collisional, magnetized plasma with fluctuations driven by a parallel ‘antenna’ current term added to Ampere's law. These fluctuations are assumed to be Alfvén waves at the relatively long wavelengths of the inertial range so that the code can represent the field fluctuation spectra over the approximate range 0.10<*k*_⊥_*ρ*_i_<100. Simulations show a break at the ion gyroscale from the 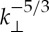 scaling of the inertial range to a 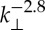 scaling for short-wavelength magnetic fluctuation spectra ([19,20,49]; figure 2). TenBarge *et al.* [50] find little ion heating, and further conclude that the electron heating rate is consistent with that of electron heating predicted by linear Landau damping. For a more complete discussion of the role of gyrokinetic simulations in the study of Alfvénic turbulence, see Howes [13].

Valentini *et al.* [51] executed 2D in-plane Vlasov simulations of short-wavelength turbulence driven by an initial spectrum of Alfvén-like fluctuations with an initial *β*_i_=0.5. The forward cascade to short wavelength is, surprisingly, a function of the electron/ion temperature ratio. At *T*_e_/*T*_i_=1, the characteristic wavevector anisotropy *k*_⊥_≫*k*_∥_ develops, but at 3<*T*_e_/*T*_i_<10 the opposite anisotropy *k*_⊥_≪*k*_∥_ arises.

In-plane 2D PIC hybrid simulations of short-wavelength turbulence initiated by the imposition of an isotropic spectrum of Alfvén waves at *kc*/*ω*_pi_<1 have been carried out with an initial *β*_i_=0.05 and *T*_e_/*T*_i_=10. In both Verscharen *et al.* [52] and Comişel *et al.* [53], the resulting forward cascades create broadband magnetic fluctuation spectra with the characteristic *k*_⊥_≫*k*_∥_ wavevector anisotropy, which is in the opposite sense of the anisotropy computed at *T*_e_/*T*_i_=10 by Valentini *et al.* [51]. Verscharen *et al.* [52] shows coupling in the short-wavelength regime not only to Alfvénic fluctuations but also to compressive modes with magnetosonic-whistler and ion Bernstein dispersion properties; Comişel *et al.* [53,54] further demonstrate coupling to a zero-frequency mode which remains the dominant component throughout their simulations. Verscharen *et al.* [52] also show that ion heating is anisotropic, with *T*_⊥i_/*T*_∥i_>1. More recently, Narita *et al.* [55] and Comişel *et al.* [56] have compared wavevector anisotropies both as observations from the *Cluster* spacecraft in the solar wind over 0.5<*β*_i_<3.6 and as results from in-plane 2D PIC hybrid simulations with initial long-wavelength Alfvén waves over 0.1<*β*_i_<2.0; both observations and simulations not only yield the characteristic *k*_⊥_≫*k*_∥_ result, but also give a uniformly decreasing value of the anisotropy factor equation (1.1) as *β*_i_ increases.

Vasquez *et al.* [57] ran 3D PIC hybrid simulations of freely decaying turbulence initiated with anisotropic Alfvénic fluctuations at *kc*/*ω*_pi_≪1 in a plasma with *β*_i_=0.10 for most runs. The proton heating rate in these simulations scales as the initial fluctuation amplitude to the power 3.2. The wavevector anisotropy tends to the characteristic *k*_⊥_≫*k*_∥_, even for modestly anisotropic initial conditions, with a scaling of the wavevector anisotropy factor (equation (1.1)) approximately proportional to *B*^2^_o_/|*δB*|^2^. Gary [58] used equations (1.2) and (1.3) with Alfvén wave kinetic linear dispersion equation solutions to argue that three-wave coupling processes should favour the development of the characteristic *k*_⊥_≫*k*_∥_ wavenumber anisotropy which is in fact demonstrated in many of the simulations. Goldstein *et al.* [44] summarize many papers reporting observational studies of Alfvén and kinetic Alfvén wave dissipation in short-wavelength turbulence in the solar wind.

### (b) *T*_⊥i_>*T*_∥i_ instabilities

Many papers describe hybrid simulations of the Alfvén-cyclotron and ion mirror instabilities driven by an ion temperature anisotropy (e.g. [59] and references therein) in both 1D and 2D configurations, showing the exponential growth of field fluctuations to saturation and the associated scattering of the anisotropic ions to yield the gradual reduction of *T*_⊥i_/*T*_∥i_. Fewer computations have displayed the temporal evolution of the fluctuating magnetic field spectra in both *k*_∥_ and *k*_⊥_ [60]. Hellinger *et al.* [61] used in-plane 2D PIC hybrid simulations and Shoji *et al.* [62] used both 2D and 3D PIC simulations to examine the competition between these two ion-anisotropy-driven instabilities. Shoji *et al.* [62] showed that, for both the Alfvén-cyclotron and the mirror instabilities, nonlinear post-saturation effects shift the enhanced fluctuations to longer wavelengths. But neither paper shows evidence for the development of broadband, turbulent-like spectra.

### (c) Interpretations and questions

(1) The gyrokinetic simulations of Alfvénic and kinetic Alfvén turbulence exclude magnetosonic-whistler fluctuations. However, hybrid and full PIC simulations can represent both incompressive and compressive modes. Such simulations should be used to address the following questions: Can Alfvénic turbulence at short wavelengths couple to magnetosonic-whistler turbulence? Can these compressive modes play a significant role in very short wavelength turbulence of the electron-scale domain?
(2) The short-wavelength Alfvénic turbulence results from the Vlasov simulation results of Valentini *et al.* [51] yield a quasi-parallel wavevector anisotropy for sufficiently large *T*_e_/*T*_i_, whereas the 2D hybrid simulations of Verscharen *et al.* [52] and Comişel *et al.* [53] at large *T*_e_/*T*_i_ produce the opposite, characteristic *k*_⊥_≫*k*_∥_ anisotropy. What is the resolution of this apparent contradiction?
(3) Can the Alfvén-cyclotron anisotropy instability enhance turbulent fluctuation spectra at short wavelengths? It is unlikely that inverse cascades from this instability would add appreciable energy to the relatively large amplitude turbulence of the inertial range, but it is possible that forward cascades from this growing mode could contribute to the physics of the short-wavelength regime.
(4) Wu *et al.* [63] carried out 2D PIC simulations with **B**_o_ out of the simulation plane, studying the decay of long-wavelength ‘Alfvén mode’ turbulence as it cascades to kinetic ion-scale lengths. At small initial amplitudes, the turbulence heats electrons primarily, but stronger turbulence leads to the domination of proton heating. This is a very interesting result which deserves further study, and particularly in 3D configurations which will allow the full consequences of Landau and cyclotron damping to become manifest.
(5) Why does the linear theory threshold of the proton mirror instability provide a better fit as a constraint to observed proton anisotropies than does the threshold of the Alfvén-cyclotron instability [64,65]?

## 5. Magnetosonic turbulence and *T*_⊥i_<*T*_∥i_ instabilities

### (a) Magnetosonic turbulence simulations

Svidzinski *et al.* [66] used a 2D PIC code with **B**_o_ in the plane of the simulation to study an initial-value magnetosonic wave turbulence cascade. The model assumes a plasma that is homogeneous, collisionless and magnetized with *β*_i_=0.10 and on which an ensemble of relatively long-wavelength (*kc*/*ω*_pi_∼0.2) fluctuations are imposed; the individual modes satisfy the predictions of kinetic linear theory for the fast (compressible) magnetosonic mode. Neither the relatively incompressible Alfvén modes nor the quasi-perpendicular ion Bernstein waves are excited to any significant degree, but the compressive fluctuations undergo a forward cascade forming a broadband spectrum of magnetosonic-whistler waves down to short wavelengths of order *kc*/*ω*_pi_∼10. In the MHD regime (*kc*/*ω*_pi_<1), the turbulence is basically isotropic, but at shorter wavelengths the cascade leads to the characteristic wavevector anisotropy in the sense of *k*_⊥_≫*k*_∥_. Svidzinski *et al.* [66] did not analyse plasma heating associated with the cascade of magnetosonic turbulence.

**Figure 3.**
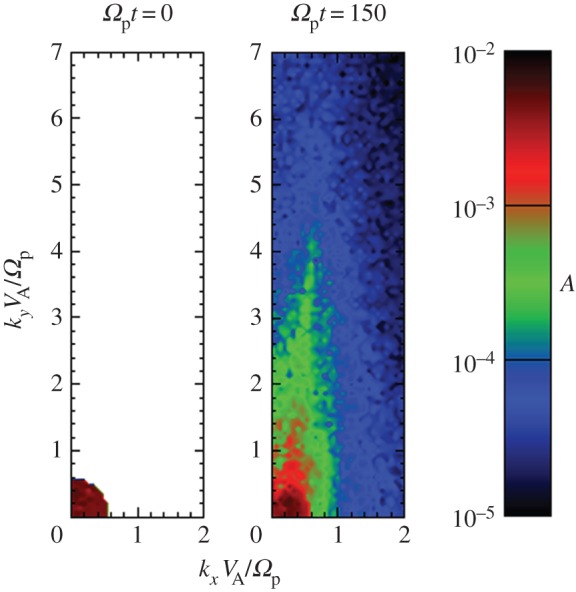
Results from a 2D PIC hybrid simulation of turbulence for an initially isotropic spectrum of magnetosonic fluctuations and *β*_i_=0.02. Here *k*_*x*_=*k*_∥_ and *k*_*y*_=*k*_⊥_. (Adapted with permission from [68].) (Online version in colour.)

Markovskii *et al.* [67] and Markovskii & Vasquez [68] carried out in-plane 2D PIC hybrid simulations with an initial *β*_*i*_=0.02 and an imposed ensemble of magnetosonic waves at *kc*/*ω*_pi_ < 1. The ensuing forward cascades yielded wavevector anisotropies with *k*_⊥_>*k*_∥_ (figure 3), and the wave energy dissipation rate increased with increasing initial fluctuation amplitudes. Two cases, both with a broad distribution of perpendicular wavevectors, were considered. For initial waves which propagated only parallel to **B**_o_, ion heating was mostly perpendicular to **B**_o_; the authors interpreted this heating as being due to the coupling of the magnetosonic waves to Bernstein modes at quasi-perpendicular propagation. For an initial spectrum of waves propagating both parallel and antiparallel to the background magnetic field, the dominant ion heating was in the parallel temperature component; the authors interpreted this result as possibly due to the nonlinear excitation of beat modes with phase speeds of the order of ion thermal speeds heating via the ion Landau resonance.

### (b) *T*_⊥i_<*T*_∥i_ instabilities

Both 1D and 2D PIC hybrid simulations of the parallel ion firehose instability [69,70] show the expected exponential growth of magnetosonic-whistler field fluctuations to eventual saturation and the associated quasi-linear scattering of ions yielding a gradual increase of *T*_⊥i_/*T*_∥i_. By contrast, 2D hybrid simulations show that the oblique ion firehose instability continues to grow after saturation of its parallel counterpart, reaching saturation by means of conversion to Alfvén waves [70]. Both instabilities scatter the ions so as to reduce the ion temperature anisotropy. The quasi-linear response of the parallel firehose means that the enhanced fluctuations of this instability maintain their narrowband, parallel propagation character, whereas the more general nonlinear response of the oblique firehose not only serves to increase *T*_⊥i_ compared with *T*_∥i_, but also leads to three-wave interactions which transfer a fraction of the enhanced fluctuation energy to *kc*/*ω*_pi_≪1 (figure 3; [70]). Nevertheless, the fluctuation spectra remain relatively narrowband in wavenumber if not in propagation direction.

### (c) Interpretations and questions

Solar wind measurements indicate that compressive magnetosonic fluctuations are much weaker than the incompressible fluctuations such as Alfvén waves which dominate inertial range plasma turbulence [71]. Nevertheless, for a full understanding of short-wavelength plasma turbulence, further computational studies of magnetosonic turbulence would be useful to answer fundamental questions such as the following:

(1) What conditions differentiate an isotropic forward cascade [66] from the anisotropic cascade [67] of magnetosonic turbulence? The anisotropic cascade is also, of course, the characteristic response for both Alfvénic and whistler turbulence, so this exception to the rule has the potential for providing fresh insights into understanding short-wavelength turbulence.
(2) Ion temperature anisotropy instabilities (as well as ion/ion kinetic instabilities discussed in ch. 8 of Gary [5]) give rise to narrowband enhanced fluctuation spectra. After instability saturation, do these enhanced spectra remain narrowband, or do they develop into broadband turbulent spectra?
(3) The linear theory threshold of the proton oblique firehose instability better constrains proton anisotropies observed in the solar wind than does the threshold of the proton parallel firehose instability [64]. Why?

## 6. Conclusion

There are two likely sources of enhanced short-wavelength electromagnetic fluctuations observed in the solar wind near 1 AU:

— Long-wavelength drivers yield broadband turbulence which cascades via nonlinear processes to dispersion and dissipation in the short-wavelength regime.
— Kinetic plasma instabilities drive narrowband enhanced fluctuations directly in the short-wavelength regime.

The purpose of this review is to summarize recent PIC cell, hybrid and gyrokinetic simulation studies which have addressed these two sources and to discuss possible relationships between them.

The fluctuations which constitute the spectra of short-wavelength magnetic fluctuations observed in the solar wind have been identified as primarily kinetic Alfvén waves [3,41,72–76], in agreement with the gyrokinetic simulations of Howes *et al.* [19,20] which show the cascade of kinetic Alfvén turbulence down to electron gyroradius wavelengths. However, there is some observational evidence that magnetosonic-whistler and/or Bernstein mode fluctuations contribute to short-wavelength turbulence in the solar wind [77–79]. In particular, the question of whether kinetic Alfvén or whistler modes are the dominant mode at electron inertial or gyro scales remains open [80,81]. Further computational studies of this question would be appropriate.

Published results to date of simulations of enhanced fluctuations at short wavelengths are consistent with the following conclusions:

— Forward cascades typically yield magnetic spectra with characteristic *k*_⊥_≫*k*_∥_ wavevector anisotropies for Alfvénic [51–57], magnetosonic [66–68] and whistler [14,15,17,27–30] fluctuations. This is the same characteristic anisotropy typically observed in the inertial range of solar wind turbulence, although some observations indicate that in the short-wavelength regime turbulence at quasi-parallel propagation is energetically dominant [82].
— Observed constraints on ion temperature anisotropies [64,65,83,84] are imposed by scattering due to enhanced field fluctuations from kinetic ion instabilities [59,64,69].
— Observed constraints on electron temperature anisotropies [45,85] are imposed by scattering due to enhanced field fluctuations from kinetic electron instabilities [37].
— Simulations of ion temperature anisotropy instabilities in the absence of long-wavelength turbulence yield enhanced field fluctuation spectra that are narrowband in both wavenumber and wavevector direction [60–62,70].
— Simulations of the whistler anisotropy instability in the absence of long-wavelength turbulence yield enhanced field fluctuation spectra that are narrowband in both wavenumber and wavevector direction [38].

In addition to the instability-specific questions stated in §§2c, 4c and 5c, there are several outstanding questions concerning the general properties of short-wavelength turbulence and kinetic instabilities:

— What are the consequences of the interaction between narrowband short-wavelength fluctuations driven by kinetic instabilities and the broadband turbulence of the inertial range?
— What are the comparative efficiencies of short-wavelength turbulence and enhanced fluctuations from kinetic instabilities in heating solar wind electrons and ions?
— Is the characteristic wavevector anisotropy in the sense of *k*_⊥_≫*k*_∥_ a universal property of forward-cascading short-wavelength turbulence, or are there conditions where more nearly isotropic fluctuations may arise?
— Osman *et al.* [86] used solar wind observations from the WIND spacecraft to show that the cascade rates of inertial range turbulence are enhanced not only for the high *β*_∥p_, high proton anisotropy regimes corresponding to kinetic instability thresholds, but also for the low *β*_∥p_, high proton anisotropy regimes where instabilities are unlikely to operate. What is the plasma physics here and does short-wavelength turbulence play a role?
— Simulations show that plasma turbulence can generate coherent structures and current sheets which lead to magnetic reconnection. Questions concerning the relationship between turbulence and reconnection are not addressed here, but have been framed in papers such as Karimabadi & Lazarian [87].
